# E2-2 Dependent Plasmacytoid Dendritic Cells Control Autoimmune Diabetes

**DOI:** 10.1371/journal.pone.0144090

**Published:** 2015-12-01

**Authors:** Lisbeth Hansen, Anja Schmidt-Christensen, Shashank Gupta, Nina Fransén-Pettersson, Tine D. Hannibal, Boris Reizis, Pere Santamaria, Dan Holmberg

**Affiliations:** 1 Department of Immunology and Microbiology, University of Copenhagen, Copehagen, Denmark; 2 Department of Experimental Medical Science, Section of Immunology, Lund University, Lund, Sweden; 3 Department of Microbiology and Immunology, Columbia Medical Center, Columbia University, New York, NY, United States of America; 4 Julia McFarlane Diabetes Research Centre (JMDRC) and Department of Microbiology, Immunology and Infectious Diseases, Snyder Institute for Chronic Diseases, University of Calgary, AB T2N 1N4, Canada; 5 Institut D’Investigacions Biomediques August Pi i Sunyer, Barcelona, Spain; Institut Pasteur, FRANCE

## Abstract

Autoimmune diabetes is a consequence of immune-cell infiltration and destruction of pancreatic β-cells in the islets of Langerhans. We analyzed the cellular composition of the insulitic lesions in the autoimmune-prone non-obese diabetic (NOD) mouse and observed a peak in recruitment of plasmacytoid dendritic cells (pDCs) to NOD islets around 8–9 weeks of age. This peak coincides with increased spontaneous expression of type-1-IFN response genes and CpG_1585_ induced production of IFN-α from NOD islets. The transcription factor E2-2 is specifically required for the maturation of pDCs, and we show that knocking out E2-2 conditionally in CD11c^+^ cells leads to a reduced recruitment of pDCs to pancreatic islets and reduced CpG_1585_ induced production of IFN-α during insulitis. As a consequence, insulitis has a less aggressive expression profile of the Th1 cytokine IFN-γ and a markedly reduced diabetes incidence. Collectively, these observations demonstrate a disease-promoting role of E2-2 dependent pDCs in the pancreas during autoimmune diabetes in the NOD mouse.

## Introduction

Type-1-diabetes (T1D) is an immune-mediated disease caused by insufficient insulin production from the pancreas. T1D as well as the spontaneous autoimmune diabetes in the non-obese-diabetic (NOD) mouse is characterized by immune cell infiltration of the pancreatic islets of Langerhans and a subsequent T-cell mediated destruction of the insulin-producing β-cells. In NOD mice, the inflammation that precedes overt diabetes is characterized by the presence of myeloid cells such as macrophages as well as dendritic cells (DCs) followed by recruitment of immune cells of lymphoid origin such as T- and B-cells [1–5]. Recently, several publications have suggested distinct and important roles for plasmacytoid DCs (pDCs) as well as type-1-IFN (IFN-I) signaling in initiation and progression of human T1D [6, 7] and diabetes of the NOD mouse model [8–10]. In contrast, pDCs have also been reported to play a regulatory role in T1D [11–13] and during progressive insulitis in animal models of diabetes [14–18]. This dual role of pDCs in autoimmune diabetes may be explained by the diverging abilities of activated pDCs to either stimulate or inhibit immune reactions by presenting antigen and producing IFN-I or by producing tolerogenic enzymes and cytokines, respectively (reviewed in [19, 20]).

In this study we have performed a detailed analysis of the cellular composition of infiltrating immune cells during progression of autoimmune diabetes. We describe that pDCs display unique kinetics of recruitment into the islets of Langerhans suggesting that this cell type plays a role in the pathogenesis. Analysis of conditional E2-2 knockout NOD mice which are defective in maturation of pDCs support this notion since pDC-deficient NOD mice display a significantly reduced expression profile of the Th1 cytokine IFN-γ during advanced insulitis and consequently a reduction in diabetes incidence.

## Results

### IFN-α-secreting pDCs peak in the pancreatic islets of NOD mice at 8–9 weeks

**Fig 1:**
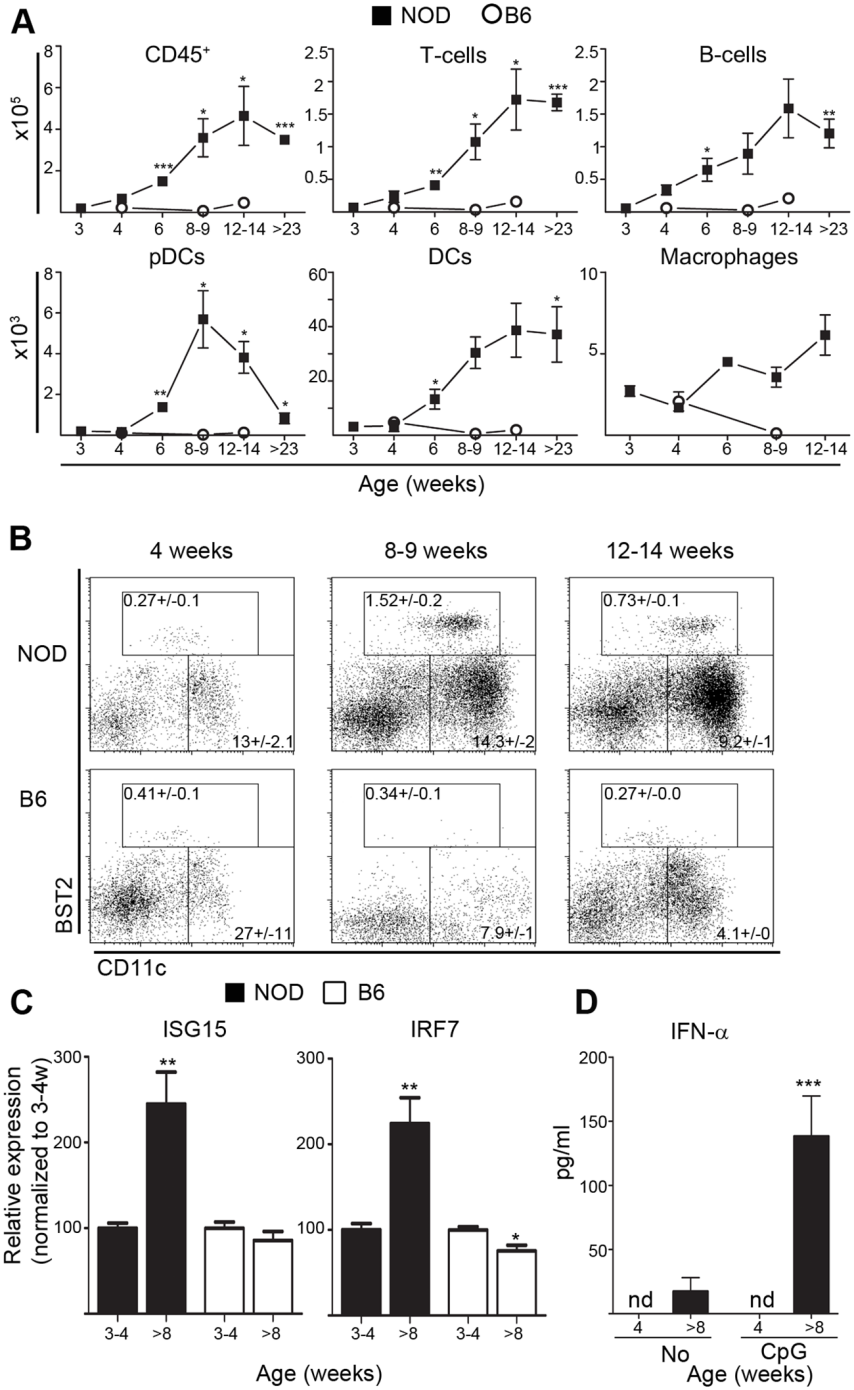
IFN-α-secreting pDCs peak in the pancreatic islets of NOD mice at 8–9 weeks. (**A**) Infiltrating leukocytes (FVD^−^CD45^+^) from pancreatic islets were analyzed by flow cytometry for total number per mouse of T-cells (CD3^+^B220^−^CD19^−^), B-cells (CD3^−^B220^+^CD19^+^), pDCs (CD3^−^CD19^−^BST2^+^CD11c^int^B220^+^), inflammatory DCs (CD3^−^CD19^−^BST2^−^CD11c^hi^MHC-II^+^) and macrophages (CD3^−^CD19^−^F4/80^+^CD11b^+^MHC-II^+^) from 3 to >23 weeks of age in NOD and B6 mice (mean ± sem, *n* = 4–21 mice, 2–5 independent experiments). * p<0.05, ** p<0.005, *** p<0.0005 compared to 4-week-old B6. (**B**) Representative dot plots of FVD^−^CD45^+^CD3^−^CD19^−^ islet cells with the percentage of pDC and inflammatory DC subsets of total CD45^+^ cells indicated (mean ± sem, *n* = 4–21 mice, 2–5 independent experiments). (**C**) Expression of interferon response genes IRF7 and ISG15 is assessed by qPCR of RNA from handpicked islets of NOD (black bars) or B6 (open bars) mice at 3 and >8 weeks (*n* = 6–11 mice, 3 independent experiments). * p<0.05, ** p<0.005. (**D**) IFN-α levels in supernatants from cultured NOD islets at 4–17 weeks of age after 40h ± TLR9 ligand CpG_1585_ is assessed by ELISA (mean ± sem, *n* = 6–23 mice, 6–9 independent experiments). nd = not detected. *** p<0.0005 compared to “No” stimulation at same age.

We isolated leukocytes from the islets of both NOD and control B6 mice at different ages and analyzed them using flow cytometry. The accumulation of recruited CD45^+^ leukocytes appeared in the NOD islets between 4–6 weeks of age (Fig 1A). From this time point we observed a gradual increase in CD45^+^ cells including T-cells (CD4^+^ and CD8^+^), B-cells, DCs, macrophages and NK-cells peaking at 12–14 weeks of age in NOD islets (Fig 1A and S1A Fig). In B6 islets no such accumulation was detected (Fig 1A and S1A Fig). The predominating cell types were of T- or B-cell origin making up more than 70% of the total CD45^+^ cells from 6 weeks of age. The decrease in CD45^+^ cells observed in islets at older ages (>23 weeks of age) was most likely due to progressive β-cell destruction resulting in reduced immune cell recruitment [21]. Together, this data concurs with previous reports analyzing NOD pancreases [1, 2, 22].

Remarkably, pDCs accumulated with a distinct peak around 8–9 weeks and then disappeared from the islets at later time points (Fig 1A and 1B). The accumulation of pDCs is accompanied by an increased IFN-I signaling confirmed by the increased expression of IRF7 (interferon response factor 7) and ISG15 (interferon stimulated gene 15) after 8–9 weeks compared to 3 week expression levels (Fig 1C) as well as increased production of IFN-α after CpG_1585_ stimulation (Fig 1D). Analysis of the IFN-I response genes in islets from B6 mice shows equal or even slightly reduced expression of both IRF7 and ISG15 from 3 weeks to >8 weeks (Fig 1C). Collectively these findings support the notion that pDCs and IFN-I signaling increase in islets of NOD mice after 8 weeks of age.

### Conditional knockout of E2-2 blocks pDC development in NOD mice

**Fig 2:**
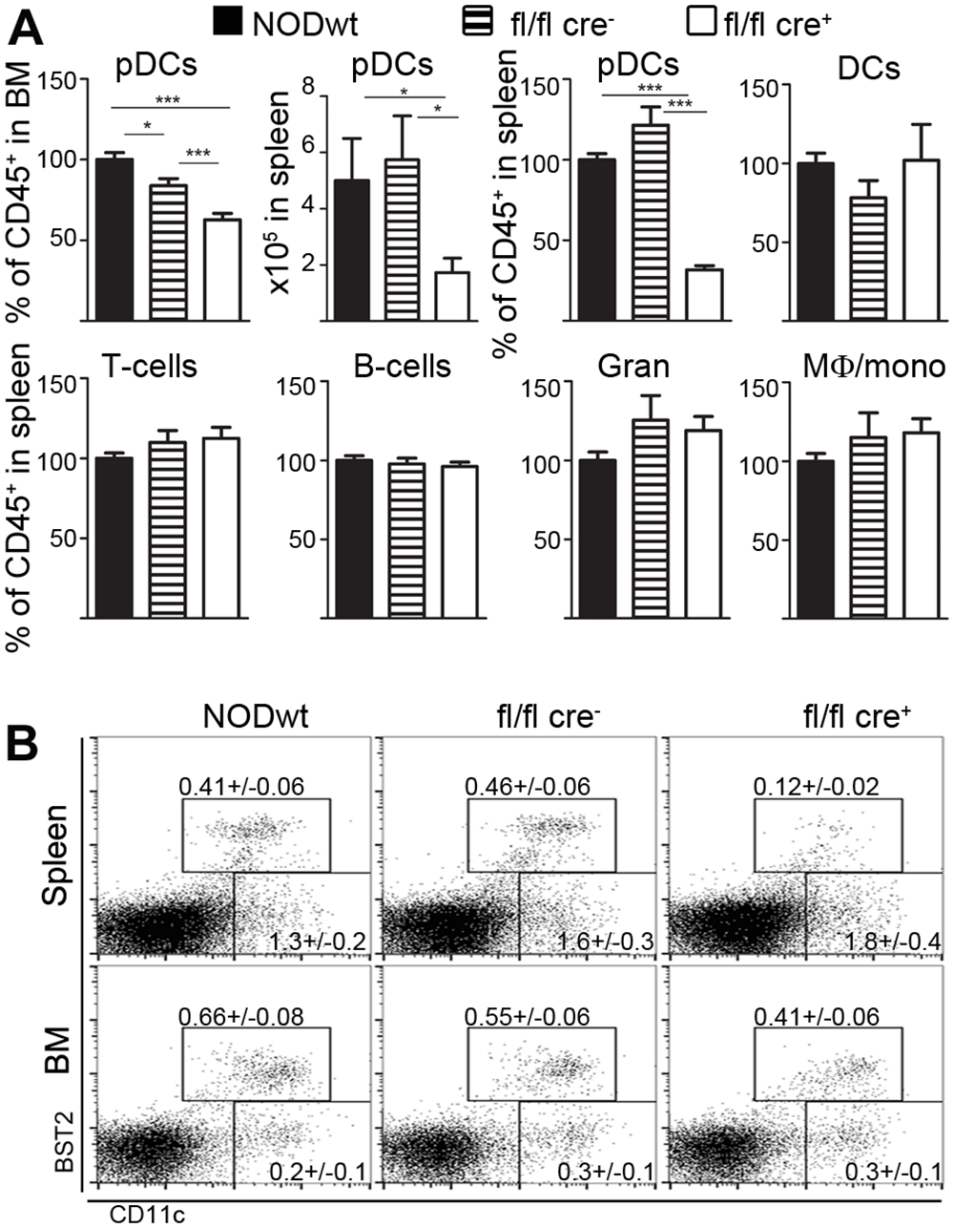
Conditional knockout of E2-2 blocks pDC development in NOD mice. (**A**) Flow cytometry analysis of BM and spleen from 8–12 weeks old NODwt (black bars), NOD.*E2-2* ^*fl/fl*^-*CD11c*.*cre* ^−^ (fl/fl cre^−^, striped bars) and NOD.*E2-2* ^*fl/fl*^-*CD11c*.*cre* ^+^ (fl/fl cre^+^, open bars) mice. Data is normalized to the level of the respective cell type in NODwt. For the pDC population the total cell number in spleen is also shown. Cell subsets include pDCs (CD3^−^CD19^−^BST2^+^CD11c^int^B220^+^), DCs (CD3^−^CD19^−^BST2^−^CD11c^hi^MHC-II^+^), T-cells (CD3^+^B220^−^CD19^−^), B-cells (CD3^−^B220^+^CD19^+^), granulocytes (Gran) (CD3^−^CD19^−^CD11b^+^SSC^hi^) and macrophages/monocytes (Mϕ/mono) (CD3^−^CD19^−^F4/80^+^CD11b^+^MHC-II^+^) (mean ± sem, *n* = 9–10 mice, 3 independent experiments). * p<0.05, ** p<0.005, *** p<0.0001. (**B**) Representative dot plots of FVD^−^CD45^+^ islet cells with percentage of pDC and inflammatory DC subsets indicated (mean ± sem, *n* = 9–10 mice, 3 independent experiments).

The specific peak in pDC recruitment to the islets and the concurrent production of IFN-α at 8–9 weeks in the NOD mice suggested that pDCs could play a decisive role in the initiation or progression of autoimmune insulitis potentially via IFN-I signaling. To directly test if pDCs were responsible for the observed IFN-α production and to investigate their potential role in the development of autoimmune diabetes, we generated transgenic NOD mice with a specific defect in pDC maturation caused by a conditional knock-out (CKO) of the E2-2 transcription factor in CD11c^+^ common DC progenitors [23, 24]. E2-2 is preferentially expressed by pDCs [23] and is only marginally involved in the development of T- and B-cell subsets [24, 25]. Similar to the previously reported B6.E2-2 CKO [23], the NOD.E2-2 CKO (fl/fl cre^+^) displayed a significant decrease in CD11c^int^ pDCs in the bone marrow and the spleen compared to NOD mice and to fl/fl cre^−^ littermate controls (Fig 2A and 2B). Impaired pDC development was consistent at all ages in spleen and peripheral LNs (S2A and S2B Fig). Other lymphoid and myeloid subsets including T-cells, B-cells, DCs, granulocytes and macrophages appeared to be unaffected (Fig 2A). In bone marrow, pDC levels are reduced to 60% of levels in NODwt in both heterozygous +/fl cre^+^ and homozygous fl/fl cre^+^ (S2A and S2E Fig). In the spleen, the effect of homozygous floxing of the E2-2 locus is more pronounced as the pDC frequency is reduced to 35% and 50% of levels in NODwt for fl/fl cre^+^ and +/fl cre^+^, respectively (S2A and S2E Fig). Both observations are consistent with our initial findings [23]. Because of inefficient recombination in precursors of pDCs, which only express CD11c intermediately, some pDCs will remain in the +/fl cre^+^ and fl/fl cre^+^ mice [26].

### Impaired recruitment of pDCs to pancreatic islets alters cytokine profile of insulitis and prevents diabetes development

**Fig 3:**
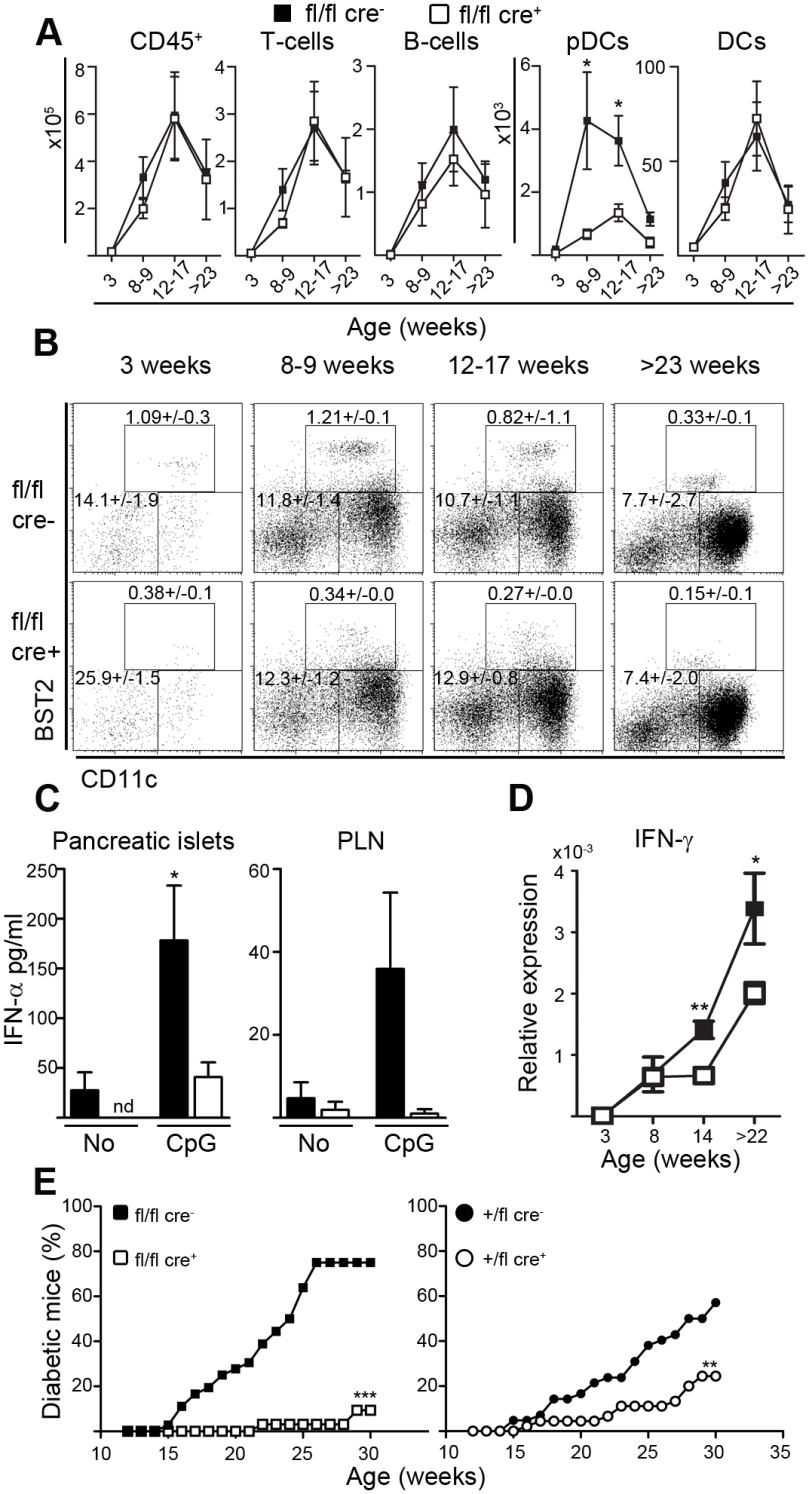
Impaired recruitment of pDCs to pancreatic islets alters cytokine profile of insulitis and prevents diabetes development. (**A**) Flow cytometry analysis of leukocytes (FVD^−^CD45^+^) from handpicked pancreatic islets of fl/fl cre^−^ (black square) or fl/fl cre^+^ (open square) mice at indicated age. Cell types include pDCs (CD3^−^CD19^−^BST2^+^CD11c^int^B220^+^), inflammatory DCs (CD3^−^CD19^−^BST2^−^CD11c^hi^MHC-II^+^), B-cells (CD3^−^ B220^+^CD19^+^), T-cells (CD3^+^B220^−^CD19^−^). Number of cells per mouse (mean ± sem, *n* = 5–15 mice, 2–5 independent experiments). * p<0.05. (**B**) Representative dot plots of FVD^−^CD45^+^CD3^−^CD19^−^ islet cells with the percentage of pDC and inflammatory DC subsets of total CD45^+^ cells indicated (mean ± sem, *n* = 5–15 mice, 2–5 independent experiments) (**C**) IFN-α levels in supernatants from cultured fl/fl cre^−^ (black bars) or fl/fl cre^+^ (open bars) islets or cultured single cell suspensions from PaLN at 8–17 weeks of age after 40h or 24h, respectively ± TLR9 ligand CpG_1585_ is assessed by ELISA (mean ± sem, *n* = 22–24 mice, 5–6 independent experiments for islets and *n* = 13–14 mice, 4–5 independent experiments for PaLN). nd = not detected. * p<0.05. (**D**) Expression of IFN-γ is assessed by qPCR of RNA from handpicked islets of fl/fl cre^−^ (black square) and fl/fl cre^+^ (open square) mice at 3->22 weeks of age (mean ± sem, *n* = 3–9 mice, 3 independent experiments). ** p<0.005, * p<0.05 (**E**) Incidence of diabetes in fl/fl cre^−^ (black square) and fl/fl cre^+^ (open square) (*n* = 36–32). *** p<0.0001. Incidence of diabetes in +/fl cre^+^ (black circle) and +/fl cre^−^ (open circle) (*n* = 45–42). ** p = 0.0014.

We next investigated if the reduction of pDCs in the NOD.E2-2 CKO mice would affect the cellular composition of insulitis as well as the cytokine profile of the infiltrated pancreatic islets. We could not detect any effect on overall insulitis score analyzed at 8–14 weeks of age (S2C Fig) or the total number of infiltrating CD45^+^ leukocytes (Fig 3A) when comparing fl/fl cre^−^ to fl/fl cre^+^. The percentage of pDCs recruited to islets was reduced at all ages in fl/fl cre^+^ mice compared to fl/fl cre^−^ (Fig 3B).

Correspondingly, the number of pDCs within pancreatic islets was clearly reduced in the fl/fl cre^+^ mice older than 8 weeks of age but not at 3 weeks of age (Fig 3A), likely reflecting the low numbers of pDCs present at the early time point. In contrast, we did not observe significant differences in the number of islet-infiltrating DCs, T- or B-cells, regulatory T-cells or in the CD4/CD8 ratio of islet infiltrating T-cells at any age (Fig 3A and S2D Fig). The cellular composition of pancreatic lymph nodes (PaLN) also remained unchanged except for the markedly reduced pDC population in the fl/fl cre^+^ mice at all ages (S2A Fig) and a slight increase in DCs most likely resulting from an effect on the common precursor of pDCs and DCs [26].

As expected, analysis of the inflammatory cytokine profile from fl/fl cre^+^ mice and littermate fl/fl cre^−^ controls revealed that CpG_1585_ induced IFN-α secretion was markedly reduced from islets isolated from the fl/fl cre^+^ mice (Fig 3C). A similar tendency was also observed in PaLN node cells after stimulation and in spontaneous IFN-α production from pancreatic islets (Fig 3C). Autoimmune diabetes of the NOD mouse is a Th1 mediated disease and IFN-γ is the main pro-inflammatory cytokine. We observed a marked reduction in the expression of IFN-γ from infiltrated pancreatic islets of fl/fl cre^+^ mice at 14 and >22 weeks of age (Fig 3D). This reduction suggests a less aggressive Th1 phenotype of the infiltrating T-cells in the scenario where pDCs are not present in the islets.

Finally, we analyzed the potential effect of the reduction of pDCs and IFN-γ expression on diabetes development. The extend of pDC reduction may control the progression to overt diabetes and therefore the diabetes incidence was determined in both homozygous fl/fl cre^+^ and heterozygous +/fl cre^+^ mice and littermate control cre^−^ mice. As illustrated in Fig 3E, a significant drop in diabetes incidence from >75% for control to <10% for fl/fl cre^+^ mice was observed. The effect on diabetes incidence was also observed in +/fl cre^+^ mice but was less pronounced in accordance with the lower reduction in peripheral pDCs in +/fl mice compared to fl/fl cre^+^.

## Discussion

Several studies have investigated the role of pDCs in the development of autoimmune diabetes and both disease promoting and inhibiting roles of this cell subset have been suggested. Diana et al. recently proposed a model in which pDCs via IFN-α interplay with neutrophils and natural antibodies to promote disease early in the development of murine T1D [8]. In that study, a peak in recruitment of pDCs to pancreatic islets is reported at 4 weeks. The significant discrepancy between these finding and the findings presented in this study could be caused by difference in the ages of mice used for analysis. Thus, while we here describe a peak in accumulation of pDCs at 8–9 weeks of age, Diana et al. do not analyze islets from mice older than 6 weeks. In our hands, pDCs and neutrophils only constitute small fractions of the total islet leukocytes at 3–4 weeks of age (Fig 1A, 1B and S1A Fig). Given the small number of CD45^+^ cells isolated from islets at this early stage we could, therefore, not confirm the early recruitment of large numbers of pDCs or neutrophils reported by Diana et al. at the same age [8]. One explanation for this discrepancy could be technical differences, such as the method of islet isolation. In the current study we handpicked islets directly after collagenase digestion minimizing the risk of contaminating islet derived leukocytes with myeloid cells from the exocrine pancreas. Alternatively, the observed discrepancy in kinetics of pDC recruitment to islets could be a result of differences between the NOD colonies. However, we find this unlikely since multiple studies performed in independent laboratories have failed to identify notable accumulation of inflammatory leukocytes in pancreatic islets of young NOD mice (analyzed in e.g. [1, 4]).

Despite this apparent absence of pDC accumulation in the pancreatic islets at 3–4 weeks, we did observe expression of interferon response genes already at this time point (Fig 1D). These observations are in line with other recent studies, which identify an early IFN-I transcriptional signature in NOD islets [4, 27], but suggest that the cellular source of IFN-α is likely to be islet resident cells at this time point.

The peak in pDC recruitment to NOD islets at 8–9 weeks of age (Fig 1A and 1B) coincide with increased CpG_1585_ induced secretion of IFN-α (Fig 1D) and increased spontaneous expression of the interferon response genes IRF7 and ISG15 (Fig 1C). The study by Diana et al. reports a peak in islet IFN-α specifically at 3 weeks of age [8]. This discrepancy could be caused by a difference in the ages of mice used for analysis, since analysis of IFN-α by Diana et al. covers islets of NOD mice at 2 to 6 weeks of age, while the present study covers IFN-α production from islets at 4 weeks and > 8 weeks of age. In addition, the 3 week peak in IFN-α observed by Diana et al. is not overlapping with the observed peak recruitment of pDCs at 4 weeks [8], suggesting that the cellular contributors to IFN-α at 3–4 weeks of age could include additional cell types.

The contribution of pDCs in T1D pathogenesis remains highly controversial and both tolerogenic and inducing roles have been linked this cell type. The present study supports an inducing role of pDCs in diabetes pathogenesis by linking reduced IFN-γ expression and lower diabetes incidence (Fig 3D and 3E) to a genetic defect in pDC maturation. This link is supported by studies of NOD mice and material from human T1D patients. Analysis of leukocytes from human T1D patients shows that mature pDCs can drive the differentiation of CD4^+^ T cells towards Th1 phenotype with increased production of IFN-γ *in vitro* in a process which is likely dependent on IFN-I [6]. Additional studies show that pDCs *in vitro* enhance auto-antigen presentation in the presence of auto-antibodies in a more efficient manner than other DC and antigen presenting cell subsets from peripheral blood of T1D patients [7]. In NOD mice, depletion of pDCs cause reduced potential of pancreatic CD8^+^ T-cells to produce IFN-γ in response to re-stimulation with IGRP_206-214_ peptide [8]. Collectively, these studies show a pDC sensitive Th1-polarization of T-cell responses causing destruction of insulin producing β-cells. Thereby pDCs control the progression from insulitis to overt diabetes. Despite the observed preventive effect on diabetes incidence, the pDC deficiency in the NOD.E2-2 CKO mice did not result in any detectable alterations in insulitis score analyzed at 8–14 weeks (S2C Fig). It is likely that analysis at time points of more advanced insulitis will show detectable differences in insulitis and β-cell mass and these studies are ongoing.

The analysis of the regulatory role of pDCs in progressive NOD insulitis demonstrates that pDCs also can provide inhibitory signals by secreting IDO [16, 17]. In the wild type NOD, the production of IDO at progressive insulitis is, however, not sufficient to control the ongoing destruction of β-cells and prevent diabetes. Moreover, a recent finding suggests that IDO expression and catalytic function is defective in pDCs from NOD mice [9]. Another set of evidence supporting a protective and regulatory role of pDCs has been obtained by analyzing induced models of diabetes [14, 15, 18]. The mechanistic evidence from these studies suggests that pDCs via TGF-β production, CXCR3 up-regulation and interplay with NKT cells and Tregs act in several ways to dampen tissue destruction.

Based on the SNP analysis of Chr 18 we cannot rule out that the alleles of *Idd21*.*1* and *Idd21*.*2*, [28, 29] located in the same chromosomal region as the *E2-2*, could be of non-NOD origin. However, since we use littermate controls in this study we can rule out that the differences observed between fl/fl cre^−^ control and fl/fl cre^+^ results from a carryover of resistance conferred by non-NOD alleles in this region of chromosome 18 or elsewhere in the genome. In addition, the integration of the Cre-recombinase transgene has previously been reported not to affect the incidence or insulitis progression after backcrossing to NOD [30].

In conclusion, this study complements previous conflicting findings of reduced diabetes incidence upon pDC depletion using antibodies [8, 10] or by blocking IFN-I signaling [31] but no effect on diabetes incidence or insulitis in NOD.IFNAR^-/-^ [27]. However, it is still unclear whether the disease-promoting role of pDCs observed here is a consequence of cytokine mediated or antigen presentation mediated activation of diabetogenic T-cells. Future studies including analysis of the particular subset of cells responding to pDC stimulation are necessary to fully understand the molecular mechanisms by which pDCs activate and enhance the diabetogenic response in NOD islets.

## Materials and Methods

### Ethics statement

Animal experiments were performed in strict accordance with the recommendations for the use of laboratory animals from the Swedish board of agriculture. The ethics committees of Umeå University and Lund University approved all animal experimental procedures (Permit number: A120-12 and M130-13). All efforts were made to minimize suffering.

### Mice

C57BL/6 (B6) and NOD mice were purchased from Taconic (Ejby, Denmark). B6.*E2-2* ^*fl/fl*^-*CD11c*.*cre* [23] were backcrossed to NOD genetic background for >10 generations to generate NOD.*E2-2* ^*fl/fl*^-*CD11c*.*cre*. SNP-based scans were used to ensure that all chromosomes, except chromosome 18 carrying the E2-2 locus were of NOD origin. By in depth SNP analysis of chromosome 18 we have limited the region flanking the E2-2 locus to <19 cM corresponding to <24 Mb (S1 Table). All animals were bred and maintained in a specific pathogen-free environment at the animal facilities at Umeå University or Lund University and all strains were maintained by continuous inbreeding. Mice were sacrificed by cervical dislocation. Only female mice were used for experiments.

### Diabetes and insulitis scoring

Female mice were monitored for diabetes incidence until 30 weeks of age by weekly measurements of urine glucose levels. Mice were considered diabetic when urine glucose was >13 mmol/l for two consecutive weeks. Preparation of pancreas sections and staining were performed as described previously [32]. Insulitis was scored in >50 islets per mouse as no infiltration (score 0), peri-insulitis (score 1), <50% of islet infiltrated (score 2) or >50% of islet infiltrated (score 3).

### Islet isolation

Pancreatic islets were isolated as previously described [3] and manually picked under stereomicroscope before further processing. Islets for flow cytometry were trypsin treated (0.05% Trypsin, 0.05% EDTA, Gibco, Waltham, MA, USA) at 37°C.

### Real-time PCR analysis

Total RNA was extracted and DNase-treated using RNeasy micro kit (Qiagen, Venlo, Netherlands). cDNA was generated using High Capacity kit (Applied Biosystems, Waltham, MA, USA). qPCR was performed using MyIQ machine (BioRad, Hercules, CA, USA) and Taqman primer/probe sets recognizing IFN-γ and interferon response genes IRF7 and ISG15 (Applied Biosystems). Expression levels are shown relative to expression of GAPDH and β-actin (Applied Biosystems).

### Flow cytometry

Single cell suspensions were prepared from spleens, bone marrow, lymph nodes (LN) or pancreatic islets by collagenase XI digestion (0.7 mg/ml, Sigma, St. Louis, MO, USA) and disaggregation through 70 μm filters and were stained at 4°C in PBS containing 2mM EDTA, 3% FCS and 0,02% NaN_3_. Blocking, surface staining and exclusion of dead cells was performed with the following Abs: α-CD45 (clone 30-F11), α-B220 (clone RA3-6B2), α-CD19 (clone eBioD3), α-F4/80 (clone BM8), α-CD49b (clone DX5), α-CD11c (clone N418), α-CD4 (clone RM.4-5), α-CD8 (clone 53–6.7), α-CD25-biotin (clone PC61.5) and Fixable Viability Dye (FVD) all from eBioscience (San Diego, CA, USA) and CD16/32 (clone 2.4G2), α-CD11b (clone M1/70), α-Ly49G2 (clone 4D11), α-TCRβ (clone H57-597) all from BD Biosciences (San Jose, CA, USA), and α-BST2 (clone 120G8.4) (Dendritics, Lyon, France). Cellular composition was analyzed using LSR II (BD Biosciences) and FlowJo software (TreeStar, Ashland, OR, USA).

### IFN-α ELISA

Single cells from PaLN (2x10^6^ cells/ml) or pancreatic islets (60 islets/well) were cultured for 24 hours in 1ml RPMI complete medium (10% FCS, 1% Pen/Strep, 0,2% Sodium Bicarbonate, 1 mM Sodium Pyruvate) (Sigma) or 40 hours in 120μl of RPMI complete medium with 5.5mM glucose, respectively. Cultures were supplemented with 5μg/ml CpG_1585_ (ODN1585, InvivoGen, San Diego, CA, USA) as indicated. Supernatants were used for IFN-α-quantification by ELISA (eBioscience). The limit of detection of IFN-α using this ELISA was determined to be 7.48 pg/ml by the producer.

### Statistical analysis

Diabetes incidence was plotted according to Kaplan-Meier method and incidence between groups compared using log-rank test. For other experiments comparison of two groups was performed using unpaired two-tailed students t-test. Data was analyzed using GraphPad Prism software (GraphPad Software, La Jolla, CA, USA).

## Supporting Information

S1 Fig Cellular composition of NOD insulitis. (A) Infiltrating leukocytes (FVD^−^CD45^+^) from pancreatic islets were analyzed by flow cytometry for total number/mouse of CD4^+^ and CD8^+^ T-cells (CD3^+^B220^−^CD19^−^), NK-cells (CD3^−^B220^−^CD49b^+^LY49G2^+^) and neutrophils (CD3^−^B220^−^CD11b^+^LY6G^+^) from 3 to >23 weeks of age in NOD and B6 mice (mean ± s.e.m., *n* = 4–21 mice, 2–5 independent experiments). * p<0.05, ** p<0.005 compared to 4-week-old B6.
(TIF)

S2 Fig Conditional knockout of E2-2 blocks pDC development in NOD mice, but does not affect insulitis score. (**A**) Flow cytometry analysis of pDC percentage among FVD^−^CD45^+^ leukocytes in spleen, PaLN or inguinal LN (ILN) from fl/fl cre^−^ (striped bars) and fl/fl cre^+^ (open bars) mice at indicated ages (mean ± s.e.m, *n* = 5–21 mice, 2–6 independent experiments). * p<0.05, ** p<0.005, *** p<0.0001. (**B**) Representative dot plots of FVD^−^CD45^+^ islet cells with percentage of pDC and inflammatory DC subsets indicated (mean ± s.e.m, *n* = 5–21 mice, 2–6 independent experiments) (**C**) Insulitis is assessed at 8–14 weeks of age in NOD.*E2-2* ^*fl/fl*^ *-CD11c*.*cre* ^−^ (fl/fl cre^−^) and NOD.*E2-2* ^*fl/fl*^ *-CD11c*.*cre* ^+^ (fl/fl cre^+^) mice (*n* = 6). Score 1 (open bars), score 2 (light grey bars), score 3 (medium grey bars), score 4 (dark grey bars). Scale bar: 100μm. (**D**) Flow cytometry analysis of T cells from islets of fl/fl cre^−^ (black) and fl/fl cre^+^ (open). Number of regulatory T cells (T reg) (FVD^−^CD45^+^TCRβ^+^CD4^+^Foxp3^+^) (*n* = 5–6 mice, 3 independent experiments) and ratio of CD4/CD8 T cells (*n* = 5–15 mice, 2–5 independent experiments) were analyzed at indicated ages. (**E**) Flow cytometry analysis of FVD^−^CD45^+^ leukocytes from BM and spleen from NODwt (black bars), and NOD.E2-2^+/fl^.CD11c.cre^+^ (+/fl cre^+^, open bars). Data is normalized to the level of the respective cell type in NODwt. Cell subsets include pDCs (CD3^−^B220^−^120G8^+^CD11c^int^B220^+^), DCs (CD3^−^B220^−^120G8^−^CD11c^hi^MHC-II^+^), T-cells (CD3^+^B220^−^CD19^−^), B-cells (CD3^−^B220^+^CD19^+^), and macrophages/monocytes (Mϕ/mono) (CD3^−^B220^−^F4/80^+^CD11b^+^MHC-II^+^) (mean ± seem, *n* = 8 mice, 2 independent experiments). * p<0.05.
(TIF)

S1 Table Microsatellite validation of B6.E22flox-CD11c.cre backcross to NOD background. Control (B6) and 2 NOD.E22flox-CD11c.cre mice at generation N5 were assayed using 82 probes to the indicated SNPs. These mice were further backcrossed for 2 generations to NOD background followed by inbreeding. Yellow indicate B6 loci and orange indicates NOD loci. Numbers in the box are the size of the expected PCR fragments.
(TIF)
