# Supplementary figures and images for: E2-2 Dependent Plasmacytoid Dendritic Cells Control Autoimmune Diabetes
Source: PLoS One. 2015 Dec 1;10(12):e0144090. doi: 10.1371/journal.pone.0144090 (PMC4666626; doi:10.1371/journal.pone.0144090)

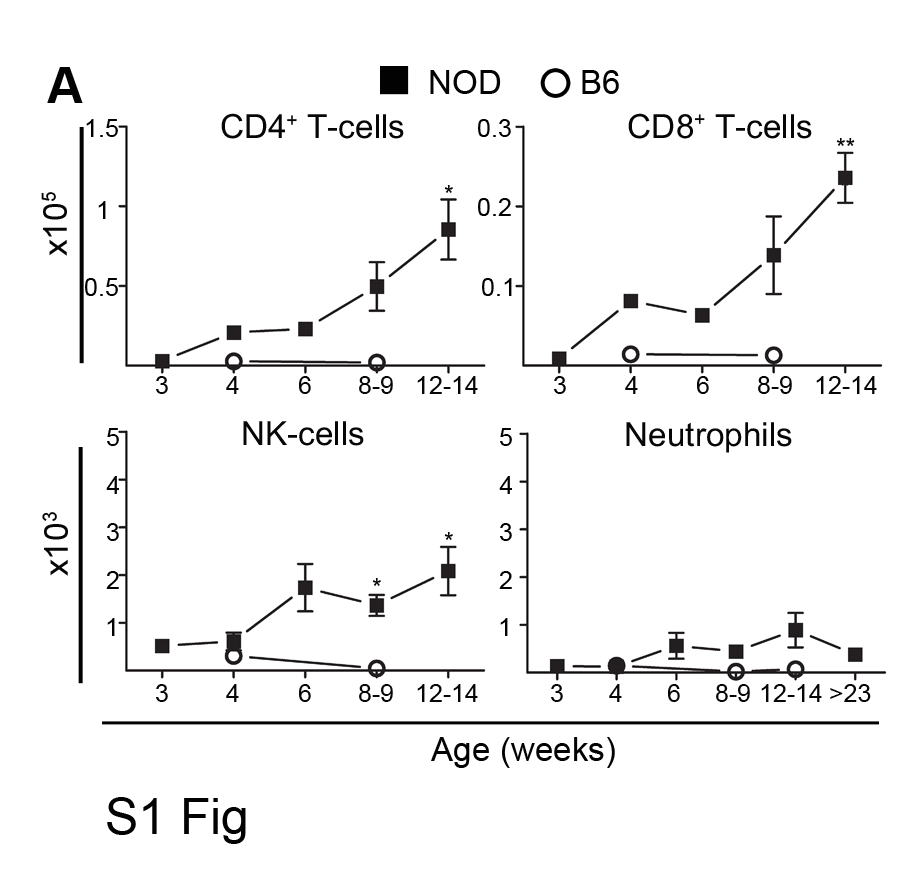

Supplement: S1 Fig — (A) Infiltrating leukocytes (FVD−CD45+) from pancreatic islets were analyzed by flow cytometry for total number/mouse of CD4+ and CD8+ T-cells (CD3+B220−CD19−), NK-cells (CD3−B220−CD49b+LY49G2+) and neutrophils (CD3−B220−CD11b+LY6G+) from 3 to >23 weeks of age in NOD and B6 mice (mean ± s.e.m., n = 4–21 mice, 2–5 independent experiments). * p<0.05, ** p<0.005 compared to 4-week-old B6. (TIF) [file pone.0144090.s001.tif]

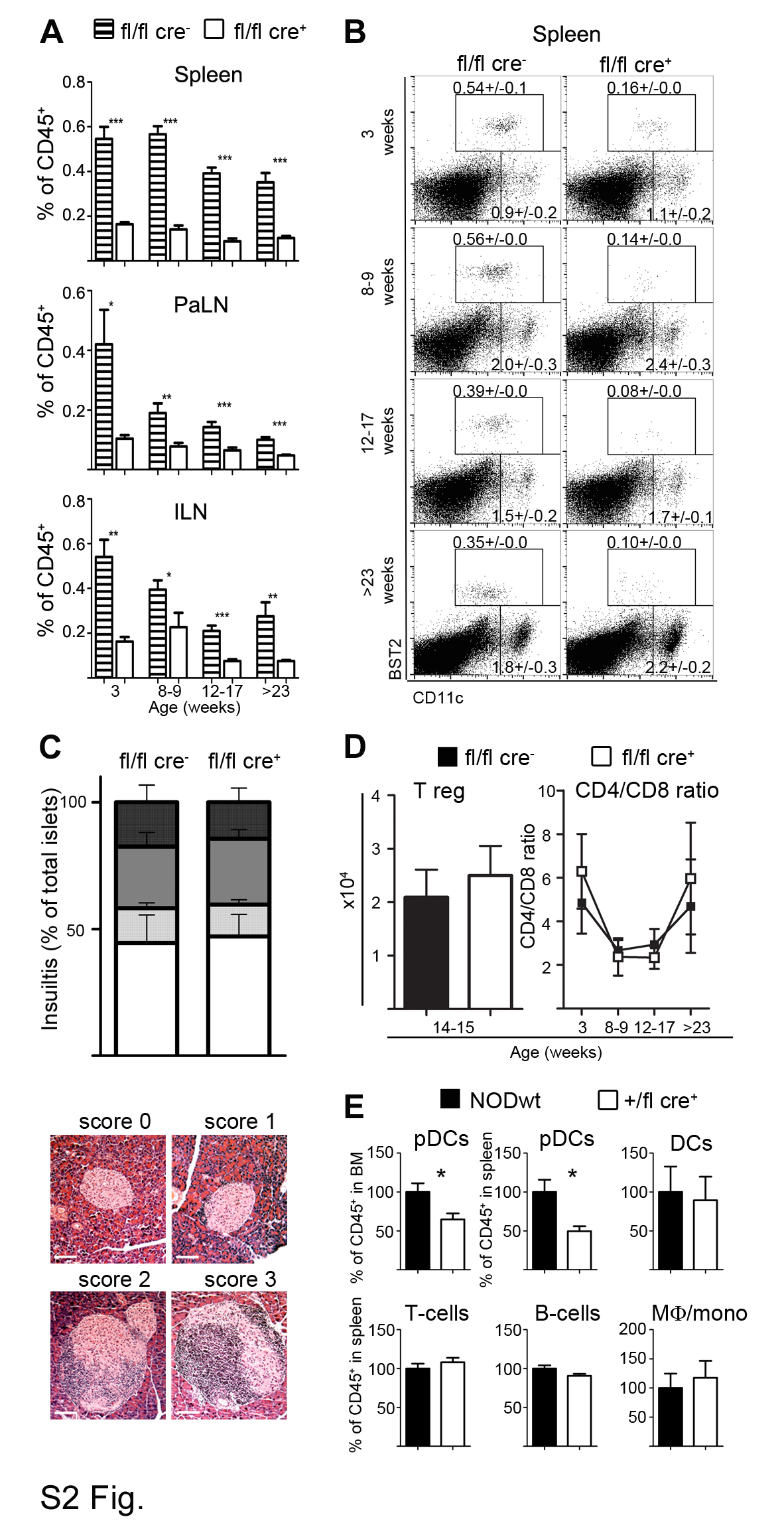

Supplement: S2 Fig — (A) Flow cytometry analysis of pDC percentage among FVD−CD45+ leukocytes in spleen, PaLN or inguinal LN (ILN) from fl/fl cre− (striped bars) and fl/fl cre+ (open bars) mice at indicated ages (mean ± s.e.m, n = 5–21 mice, 2–6 independent experiments). * p<0.05, ** p<0.005, *** p<0.0001. (B) Representative dot plots of FVD−CD45+ islet cells with percentage of pDC and inflammatory DC subsets indicated (mean ± s.e.m, n = 5–21 mice, 2–6 independent experiments) (C) Insulitis is assessed at 8–14 weeks of age in NOD.E2-2 fl/fl -CD11c.cre − (fl/fl cre−) and NOD.E2-2 fl/fl -CD11c.cre + (fl/fl cre+) mice (n = 6). Score 1 (open bars), score 2 (light grey bars), score 3 (medium grey bars), score 4 (dark grey bars). Scale bar: 100μm. (D) Flow cytometry analysis of T cells from islets of fl/fl cre− (black) and fl/fl cre+ (open). Number of regulatory T cells (T reg) (FVD−CD45+TCRβ+CD4+Foxp3+) (n = 5–6 mice, 3 independent experiments) and ratio of CD4/CD8 T cells (n = 5–15 mice, 2–5 independent experiments) were analyzed at indicated ages. (E) Flow cytometry analysis of FVD−CD45+ leukocytes from BM and spleen from NODwt (black bars), and NOD.E2-2+/fl.CD11c.cre+ (+/fl cre+, open bars). Data is normalized to the level of the respective cell type in NODwt. Cell subsets include pDCs (CD3−B220−120G8+CD11cintB220+), DCs (CD3−B220−120G8−CD11chiMHC-II+), T-cells (CD3+B220−CD19−), B-cells (CD3−B220+CD19+), and macrophages/monocytes (Mϕ/mono) (CD3−B220−F4/80+CD11b+MHC-II+) (mean ± seem, n = 8 mice, 2 independent experiments). * p<0.05. (TIF) [file pone.0144090.s002.tif]

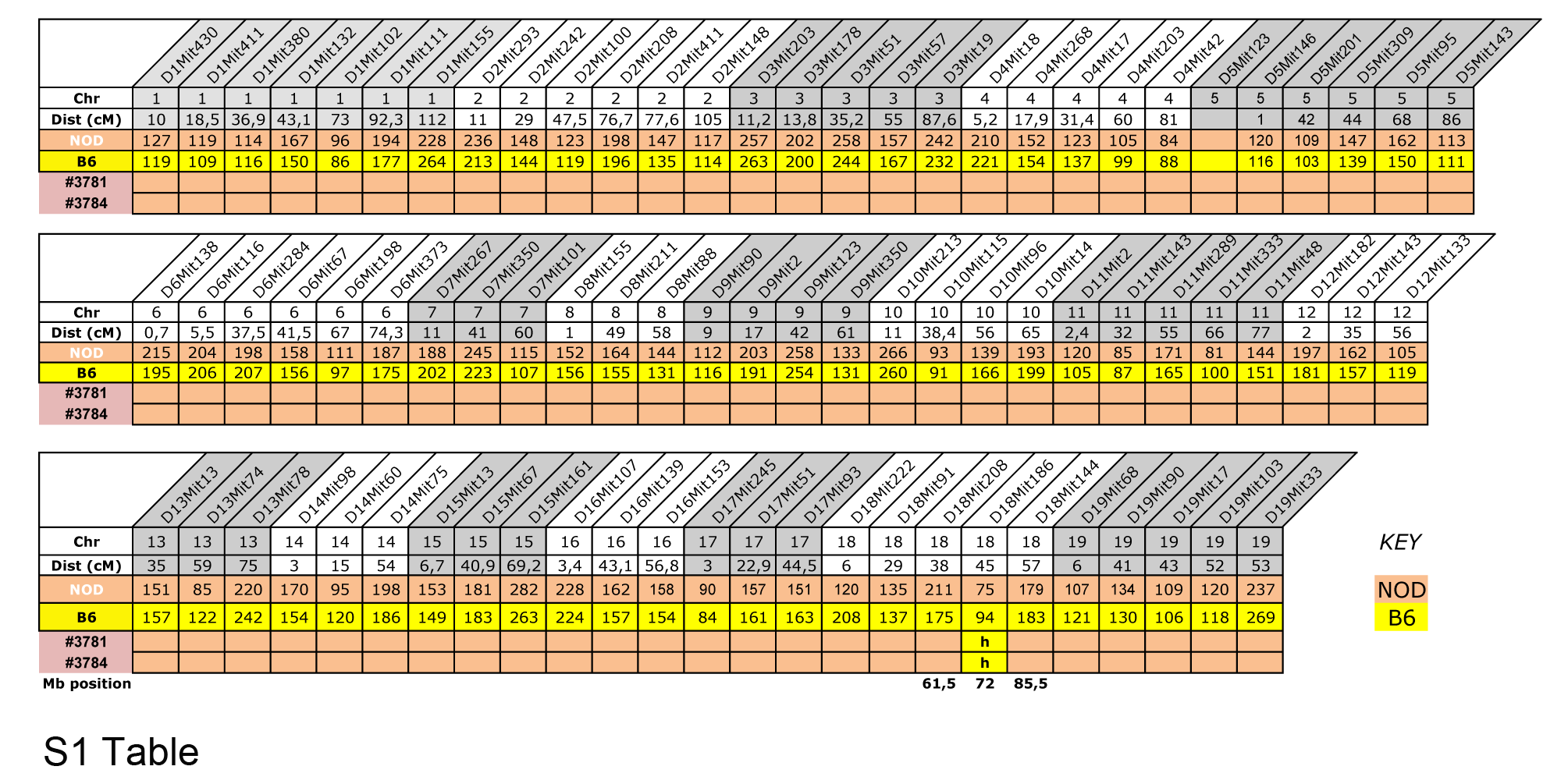

Supplement: S1 Table — Control (B6) and 2 NOD.E22flox-CD11c.cre mice at generation N5 were assayed using 82 probes to the indicated SNPs. These mice were further backcrossed for 2 generations to NOD background followed by inbreeding. Yellow indicate B6 loci and orange indicates NOD loci. Numbers in the box are the size of the expected PCR fragments. (TIF) [file pone.0144090.s003.tif]
